# Effects and Mechanisms of Probiotics, Prebiotics, Synbiotics, and Postbiotics for the Prevention and Management of Alzheimer’s Disease: A Narrative Review

**DOI:** 10.3390/antiox15030347

**Published:** 2026-03-10

**Authors:** Ting Chen, Haoqi Chen, Yingzhen Qiu, Yixiao Liu, Mengxing Xie, Siyu Huang, Kaiying Feng, Jie Zhuang, Lu Chen, Yanming Chen, Huabin Li, Mengtao Yang, Zhijun Yang, Huilian Zhu

**Affiliations:** 1 Guangdong Provincial Key Laboratory of Food, Nutrition and Health, School of Public Health, Sun Yat-sen University, Guangzhou 510080, China; chent279@mail2.sysu.edu.cn (T.C.); chenhq55@mail2.sysu.edu.cn (H.C.); qiuyzh8@mail2.sysu.edu.cn (Y.Q.); liuyx596@mail2.sysu.edu.cn (Y.L.); xiemx36@mail2.sysu.edu.cn (M.X.); huangsy9@alumni.sysu.edu.cn (S.H.); fengky5@mail2.sysu.edu.cn (K.F.); zhuangj26@mail2.sysu.edu.cn (J.Z.); chenlu66@mail2.sysu.edu.cn (L.C.); lihuabin@mail.sysu.edu.cn (H.L.); yangmt27@mail.sysu.edu.cn (M.Y.); 2 Institute of Fruit Tree Research, Guangdong Academy of Agricultural Sciences, Key Laboratory of South Subtropical Fruit Biology and Genetic Resource Utilization, Ministry of Agriculture and Rural Affairs, Guangdong Provincial Key Laboratory of Science and Technology Research on Fruit Tree, Guangzhou 510640, China; 3 Department of Endocrinology and Metabolic Diseases, The Key Laboratory of Diabetes Prevention and Treatment in Guangdong Province, The Eighth Affiliated Hospital of Sun Yat-sen University, Shenzhen 518033, China; chyanm@mail.sysu.edu.cn; 4 Department of Endocrinology and Metabolic Diseases, The Third Affiliated Hospital of Sun Yat-sen University, Guangzhou 510630, China

**Keywords:** Alzheimer’s disease, probiotics, prebiotics, synbiotics, postbiotics, microbiota–gut–brain axis

## Abstract

Alzheimer’s disease (AD) is a rapidly escalating global health crisis with limited effective treatments. Emerging research underscores the pivotal role of the microbiota–gut–brain axis in AD pathogenesis, prompting the exploration into gut microbiota-targeted interventions. This narrative review aimed to comprehensively synthesize the latest epidemiological, experimental, and clinical evidence regarding the effects and mechanisms of probiotics, prebiotics, synbiotics, and postbiotics (PPSPs) in AD prevention and management. We conducted a narrative review of relevant literature from the Web of Science and PubMed databases. The search focused on articles published within the last 5 years using keywords such as “Alzheimer’s disease”, “AD”, “gut-brain axis”, “gut microbiota”, “probiotics”, “prebiotics”, “synbiotics”, and “postbiotics”. The findings suggest that PPSPs mitigate AD pathology and improve cognitive performance by modulating gut microbiota, strengthening intestinal barrier integrity, decreasing amyloid-beta (Aβ) deposition and tau hyperphosphorylation, reducing neuroinflammation and oxidative stress, regulating neurotransmitter metabolism, and promoting synaptic plasticity. Some studies also report varied outcomes, attributable to factors like strain specificity, dosage, intervention duration, patient heterogeneity, and methodological differences. In conclusion, targeting the microbiota–gut–brain axis with PPSPs offers a promising, mechanism-based strategy for AD, though further research is essential to optimize specific interventions for clinical application.

## 1. Introduction

The global demographic landscape is undergoing profound changes, with the number and proportion of individuals aged 60 and above steadily increasing [1]. In 2020, this age group reached 1 billion, and is projected to grow to 1.4 billion by 2030 and 2.1 billion by 2050 [1]. This demographic shift is closely associated with the increasing incidence of age-related health conditions, with Alzheimer’s disease (AD) being a prominent concern [2]. According to the World Health Organization, AD is the most common form of dementia and contributes to 60% to 70% of the 57 million cases globally [3]. There are nearly 10 million new cases of dementia diagnosed every year [3]. In 2019, the global economic cost of dementia was estimated at 1.3 trillion US dollars [3]. Women are disproportionately affected by the disease and provide approximately 70% of informal care hours globally [3,4]. As the seventh leading cause of mortality and one of the major causes of disability and dependency among older people globally, AD and other dementias killed 1.8 million lives in 2021 [4]. By 2025, it is projected that approximately 7.2 million Americans aged 65 and older will be affected by AD [5]. Although existing pharmacological treatments, such as lecanemab and donepezil, offer symptomatic management or slightly delay disease progression, they are frequently associated with adverse effects [6]. Concurrently, non-pharmacological interventions for AD, such as physical activity and reminiscence therapy, provide alternative approaches; however, these methods often face challenges in long-term compliance and generally do not address the underlying pathological processes of the disease [6]. Therefore, there is a pressing demand for safe, economical, and mechanism-based interventions to combat AD.

In recent years, a rapidly emerging field has focused on the gut microbiota and its intricate bidirectional communication network with the central nervous system, often referred to as the “microbiota-gut-brain axis” or simply the “gut-brain axis” [7]. The gut microbiota directly and indirectly shapes brain function and cognitive health via bioactive metabolites, immune modulation, neuroendocrine signaling, and maintenance of intestinal and blood–brain barrier (BBB) integrity [8]. Accumulating evidence increasingly links gut microbial dysbiosis to AD onset and progression, reporting altered microbiota profiles in patients characterized by decreased diversity and shifted abundance of specific genera, including *Bifidobacterium* and *Bacteroides* [9]. Against this backdrop, targeting the gut microbiota is being explored as a promising strategy for AD using probiotics, prebiotics, synbiotics, and postbiotics (PPSPs) [10,11,12,13]. According to the International Scientific Association for Probiotics and Prebiotics (ISAPP) consensus, probiotics are live microorganisms that provide health benefits to the host [10]. Prebiotics are substrates selectively used by host microorganisms for health benefits [11]. Synbiotics combine live microorganisms and substrates to collectively improve health [12]. Postbiotics consist of preparations of inanimate microorganisms or their components that confer health benefits [13]. PPSP interventions could mitigate AD hallmarks by suppressing neuroinflammation and decreasing the deposition of amyloid-beta (Aβ) and tau proteins [14,15,16]. Moreover, PPSPs strengthen the BBB and enhance synaptic plasticity via the production of neuroprotective metabolites like short-chain fatty acids (SCFAs) [14,15,16]. Overall, these interventions might help restore gut–brain homeostasis and effectively attenuate the progression of cognitive decline [14,15,16].

Accordingly, this narrative review synthesizes recent and high-quality literature focusing on the effects and mechanisms of PPSPs in the prevention and management of AD. This review offers a comprehensive and integrated assessment of the most up-to-date evidence from epidemiological, experimental, and clinical studies on PPSPs in AD, delineating their diverse mechanisms of action. Ultimately, this review aims to contribute to a better understanding of PPSPs’ potential adjunctive role in AD, informing the development of future clinical applications.

## 2. Methods

This narrative review was conducted based on three main steps: literature search, content screening, and results synthesis. We searched the Web of Science and PubMed databases to identify relevant studies. The search was completed in August 2025 and focused on English-language articles published between January 2019 and August 2025. We used the following Boolean search strings: (“Alzheimer’s disease” OR “AD” OR “Alzheimer”) AND (“gut-brain axis” OR “gut microbiota”) AND (“probiotics” OR “prebiotics” OR “synbiotics” OR “postbiotics” OR “PPSP”). After the initial search, we removed duplicate records. We then reviewed the titles and abstracts to ensure the studies met the inclusion criteria. The inclusion criteria focused on peer-reviewed original research, including epidemiological investigations, in vitro cellular studies, in vivo animal experiments, and human clinical trials. Exclusion criteria included conference abstracts, editorials, and non-English publications. Two reviewers independently screened the titles and abstracts of the retrieved records. Any conflicts regarding study eligibility were resolved through discussion and consensus. The selected literature included epidemiological, experimental, and clinical evidence regarding the effects and mechanisms of PPSPs on AD. Finally, we summarized these findings to compose this review. As this is a narrative review, the literature search did not need to be recorded on any specific platform, and the flowchart of the literature search is not needed [17,18,19].

## 3. Pathogenesis of Alzheimer’s Disease

AD, as a progressive neurodegenerative disorder and the leading cause of dementia globally, is characterized by distinctive neuropathological hallmarks [20]. These cardinal features include the extracellular deposition of amyloid plaques, primarily composed of aggregated Aβ protein (particularly Aβ_42_), and the intracellular accumulation of neurofibrillary tangles comprised of hyperphosphorylated tau assemblies [20]. In addition, AD pathologies also include persistent microglial activation, reactive astrogliosis, and the consequent chronic neuroinflammation, together with synaptic dysfunction, neuronal loss, BBB disruption (compromising brain homeostasis), and decreased cerebral glucose metabolism (the brain’s main fuel) [21]. Collectively, these pathological signatures culminate in substantial synaptic and neuronal attrition, ultimately manifesting as macroscopic brain atrophy and gradual clinical progression of cognitive and behavioral deficits [22].

**Figure 1:**
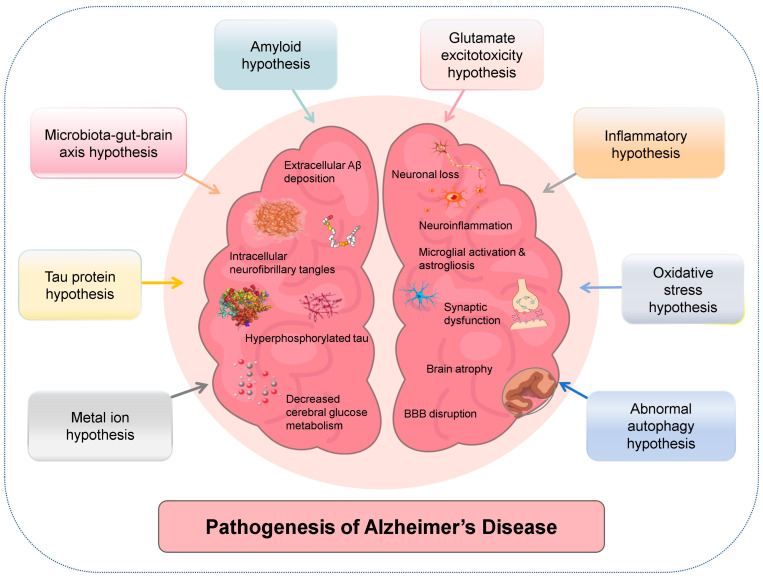
Pathogenesis of Alzheimer’s disease. This figure illustrates the multifaceted pathogenesis and core pathological hallmarks of Alzheimer’s disease. Various proposed pathogenic hypotheses, such as the amyloid hypothesis, tau protein hypothesis, microbiota–gut–brain axis hypothesis, oxidative stress hypothesis, glutamate excitotoxicity hypothesis, inflammatory hypothesis, metal ion hypothesis, and abnormal autophagy hypothesis, contribute to Alzheimer’s disease progression. These pathways lead to key Alzheimer’s disease pathologies such as extracellular Aβ deposition, intracellular neurofibrillary tangles composed of hyperphosphorylated tau assemblies, synaptic dysfunction, neuronal loss, neuroinflammation (including microglial activation and astrogliosis), decreased cerebral glucose metabolism, brain atrophy, and BBB disruption. Abbreviations: Aβ, amyloid-β; BBB, blood–brain barrier.

The etiology of AD is complex and multifaceted, and its precise pathogenic mechanisms are yet to be fully elucidated [23]. Currently, research on AD pathogenesis is primarily based on several interconnected theoretical frameworks, with no definitive conclusions yet established [23]. The amyloid cascade hypothesis posits that extracellular deposition of Aβ peptides is the initiating event [24], whereas the tau protein hypothesis emphasizes intraneuronal tau hyperphosphorylation as the proximal driver of neurodegeneration [25]. Recent network-based models integrate these two views by demonstrating a bidirectional Aβ-tau toxic axis, in which oligomeric Aβ_42_ accelerates tau seeding and, conversely, pathological tau impairs Aβ clearance [23]. This protein-centric pathology is intimately connected to the inflammatory hypothesis, which posits that chronic activation of microglia and astrocytes sustains an inflammatory milieu, further exacerbating neuronal damage [26]. Concomitantly, the oxidative stress hypothesis underscores the detrimental role of excessive reactive oxygen species (ROS) production, which compromises cellular constituents and mitochondrial efficiency [27]. Moreover, the glutamate excitotoxicity hypothesis suggests that dysregulated glutamate signaling causes neuronal injury via calcium overload, significantly affecting cognitive faculties [28]. The metal ion hypothesis further implicates imbalances in essential metal ions, such as iron and copper, in accelerating Aβ aggregation and oxidative damage [29], while the abnormal autophagy hypothesis highlights deficiencies in cellular waste removal pathways, contributing to the accumulation of pathological proteins [30]. Furthermore, complex interactions within the microbiota–gut–brain axis (microbiota–gut–brain axis hypothesis) also contribute to the pathogenesis of AD [23]. Most importantly, these diverse hypotheses are not isolated but rather form a tightly integrated network, where each factor can mutually influence and perpetuate others, collectively propelling the progressive neurodegeneration characteristic of AD [23] (Figure 1).

## 4. Effects and Mechanisms of Probiotics on Alzheimer’s Disease

**Figure 2:**
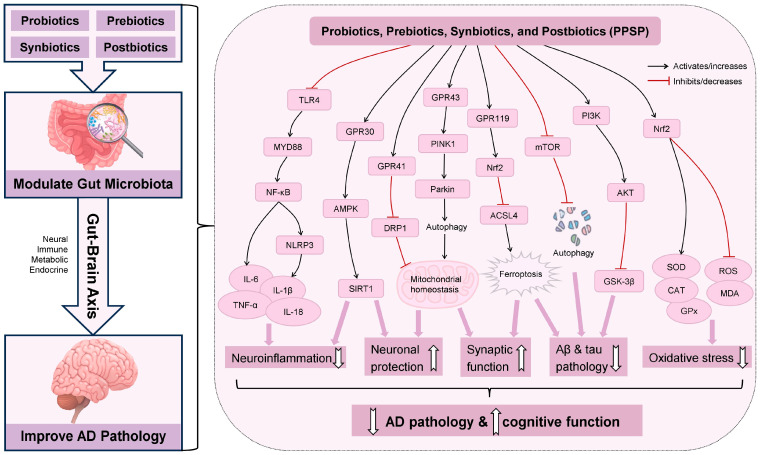
Effects and mechanisms of probiotics, prebiotics, synbiotics, and postbiotics on AD via the gut–brain axis. This figure illustrates the multifaceted mechanisms by which probiotics, prebiotics, synbiotics, and postbiotics (PPSPs) modulate AD pathogenesis through the gut–brain axis. PPSPs exert their effects by modulating gut microbiota, thereby influencing brain function via the gut–brain axis, which integrates neural, immune, metabolic, and endocrine signaling pathways. The main effects and mechanisms of PPSPs on AD include the following: (1) PPSPs could mitigate neuroinflammation by inhibiting TLR4/MYD88/NF-κB/NLRP3 pathway, characterized by decreased levels of pro-inflammatory cytokines (IL-1β, IL-6, IL-18, and TNF-α). (2) PPSPs could promote neuronal protection by activating GPR30/AMPK/SIRT1 pathway. (3) PPSPs could maintain mitochondrial homeostasis through downregulation of DRP1 via GPR41 and activation of PINK1/Parkin-mediated mitophagy via GPR43, thereby improving neuronal protection and synaptic function. (4) PPSPs could reduce Aβ deposition and restore synaptic function by inhibiting ferroptosis through the activation of GPR119 and Nrf2, which subsequently inhibit ACSL4 expression. (5) PPSPs could ameliorate Aβ pathology by promoting autophagy through the inactivation of the mTOR signaling pathway. (6) PPSPs could mitigate Aβ and tau pathology by activating the PI3K/AKT/GSK3β pathway. (7) PPSPs could alleviate oxidative stress by activating Nrf2 pathway, evidenced by reduced levels of ROS and MDA, concurrent with elevated levels of SOD, CAT, and GPx. Collectively, these actions mitigate AD pathology and improve cognitive function. Abbreviations: Aβ, amyloid-β; ACSL4, acyl-CoA synthetase long-chain family member 4; AD, Alzheimer’s disease; AKT, protein kinase B; AMPK, AMP-activated protein kinase; CAT, catalase; DRP1, dynamin-related protein 1/mitochondrial fission protein; GPR30, G-protein-coupled receptor 30; GPR41, G-protein-coupled receptor 41; GPR43, G-protein-coupled receptor 43; GPR119, G-protein-coupled receptor 119; GPx, glutathione peroxidase; GSK-3β, glycogen synthase kinase 3β; IL-1β, interleukin-1β; IL-6, interleukin-6; IL-18, interleukin-18; MDA, malondialdehyde; mTOR, mechanistic target of rapamycin; MYD88, myeloid differentiation primary response 88; NF-κB, nuclear factor-κB; NLRP3, NOD-like receptor family pyrin domain containing 3; Nrf2, nuclear factor erythroid 2-related factor 2; Parkin, Parkin RBR E3 ubiquitin protein ligase; PI3K, phosphoinositide 3-kinase; PINK1, PTEN-induced kinase 1; PPSPs, probiotics, prebiotics, synbiotics, and postbiotics; ROS, reactive oxygen species; SIRT1, sirtuin 1; SOD, superoxide dismutase; TLR4, toll-like receptor 4; TNF-α, tumor necrosis factor-α.

The use of probiotics to prevent and manage AD has recently attracted significant attention. Effects and mechanisms of probiotics on AD are summarized and shown in Figure 2 and Table 1 and Table 2, which will be discussed in detail below.

### 4.1. Experimental Studies

Experimental studies suggested that specific probiotics may mitigate AD pathology by modulating the gut microbiota and interacting with the gut–brain axis. A growing number of experimental studies have suggested the benefits of single-strain probiotics in mitigating AD pathology. For instance, *Lactobacillus plantarum* MA2, derived from Tibetan kefir grains, effectively mitigated cognitive impairment in D-galactose/AlCl_3_-induced AD rats by regulating the gut microbiota and glycometabolism, and by mitigating neuronal damage and Aβ deposition [31]. Moreover, *Lactobacillus plantarum* MA2 mitigated neuroinflammation by inhibiting microglial activation and the Toll-like receptor 4 (TLR4)/myeloid differentiation primary response 88 (MYD88)/NOD-like receptor family pyrin domain containing 3 (NLRP3) signaling pathway [31]. Similarly, *Lactobacillus plantarum* DP189 administration effectively elevated levels of dopamine, γ-aminobutyric acid (GABA), and serotonin (5-HT), mitigated neuronal injury and Aβ accumulation, and averted cognitive decline in D-galactose/AlCl_3_-induced AD model mice, while curbing tau hyperphosphorylation by modulating the phosphatidylinositol 3-kinase (PI3K)/protein kinase B (AKT)/glycogen synthase kinase-3β (GSK-3β) pathway and the microbiota–gut–brain axis [32]. Furthermore, a study indicated that layer-by-layer encapsulation safeguarded probiotics from gastrointestinal damage, and this encapsulated *Lactiplantibacillus plantarum* alleviated brain neuroinflammation, neuronal damage, tau phosphorylation, Aβ accumulation, and intestinal barrier integrity impairment, and bolstered synaptic plasticity by normalizing the intestinal microbiota balance in transgenic mice overexpressing human amyloid precursor protein with the Swedish mutation (APPswe) and presenilin-1 (PS1) with the M146L mutation (APP/PS1 mice) [33]. Additionally, a 12-week combined intervention of memantine and *Lactobacillus plantarum* in choline-treated APP/PS1 mice significantly improved cognitive function by reducing hippocampal Aβ levels, protecting neurons, and decreasing trimethylamine N-oxide (TMAO) synthesis and neuroinflammation, likely through gut microbiota modulation [34].

*Bifidobacterium* strains also showed significant promise in attenuating AD pathology through diverse mechanisms. For example, *Bifidobacterium breve* MCC1274 was demonstrated to attenuate AD-related pathologies in C57BL/6J mice by activating the AKT/GSK-3β pathway, enhancing synaptic protein levels and reducing tau phosphorylation, Aβ_42_ levels, and neuroinflammation [35]. Similarly, this probiotic might alleviate memory deficits in *App^NL-G-F^* mice via an amyloid-cascade-independent mechanism by reducing tau hyperphosphorylation and chronic stress, and by further augmenting synaptic protein levels and neuronal activity in the hippocampus [36]. Moreover, both *Bifidobacterium breve* WX and CCFM1025 notably strengthened synaptic plasticity and elevated the levels of postsynaptic density protein 95 (PSD-95), fibronectin type III domain-containing protein 5 (FNDC5), and brain-derived neurotrophic factor (BDNF) in Aβ_1–42_-treated mice by regulating the gut microbiome [37]. Integrative metabolome analysis showed that *Bifidobacterium breve* CCFM1025 intervention effectively reversed the disrupted metabolite profile caused by Aβ injection, with altered metabolites mainly involved in amino acid metabolism [38]. Additionally, *Bifidobacterium breve* HNXY26M4 alleviated cognitive impairment, synaptic dysfunction, and neuroinflammation in APP/PS1 mice by reshaping the gut microbial community and modulating SCFAs levels [39]. Furthermore, six months of *Bifidobacterium longum* 1714 intervention in APP/PS1 mice curbed cortical and hippocampal Aβ accumulation, restrained microglial activation, and lessened the release of interferon (IFN)-γ, interleukin (IL)-1β, IL-6, and tumor necrosis factor (TNF)-α, thereby alleviating AD-related pathology [40]. Another study showed that *Bifidobacterium lactis* Probio-M8, extracted from human breast milk, mitigated Aβ plaque burden and cognitive deficits in APP/PS1 mice by restoring gut microbial homeostasis [41]. *Bifidobacterium lactis* CBT BL3 was also found to mitigate memory impairment in mice with Aβ-induced cognitive deficits by downregulating apoptosis-related proteins and abnormal activation of mitogen-activated protein kinases (MAPKs), including p38 MAPK, c-Jun N-terminal kinase (JNK), and extracellular signal-regulated kinase (ERK) 1/2 [42].

*Akkermansia muciniphila*, *Clostridium butyricum*, and *Saccharomyces boulardii* have also shown remarkable benefits in attenuating AD. Specifically, in high-fat diet-fed APP/PS1 mice, *Akkermansia muciniphila* treatment notably improved glucose tolerance, restored intestinal barrier function, ameliorated dyslipidemia, reduced cortical Aβ_40_ and Aβ_42_ levels, and alleviated cognitive impairment [43]. In addition, *Clostridium butyricum* treatment markedly improved cognitive performance in APP/PS1 mice via the gut–brain axis [44]. It reduced the Aβ plaque burden, microglial activation, and the release of IL-1β and TNF-α, thereby suppressing microglia-driven neuroinflammation [44]. Furthermore, *Saccharomyces boulardii* mitigated cognitive impairment in AD mice through the gut–brain axis, with restoration of fungal microbiota homeostasis and concurrent suppression of neuroinflammation [45].

In addition to single-strain probiotics, multi-strain probiotics have shown promise in alleviating AD-like cognitive impairment. In senescence-accelerated prone 8 (SAMP8) mice administered probiotic-2 (containing *Lactobacillus rhamnosus* and *Bifidobacterium lactis*) or probiotic-3 (containing *Lactobacillus rhamnosus*, *Lactobacillus acidophilus*, and *Bifidobacterium lactis*) for 8 weeks, cognitive deficits, Aβ/tau pathology, neuroinflammation and neural injury were markedly attenuated via the AKT/GSK-3β phosphorylation pathway [47]. Additionally, a probiotic cocktail, including five *Enterococcus* and five *Lactobacillus* strains derived from the infant gut, decreased AD pathology biomarkers, including Aβ accumulation, microglial activity, and neuroinflammation, and maintained the integrity of BBB tight junctions by modulating the inflammatory microbiome in APP/PS1 mice [48]. Meanwhile, the novel probiotic formulation BIOCG, comprising *Bifidobacterium animalis* subsp. *lactis*, *Bifidobacterium longum*, and *Lactobacillus plantarum*, increased microbial diversity and mature dendritic spine density and alleviated neuroinflammation in 3xTg and 3xTg; Thy1-YFP AD mice, thereby attenuating AD and preserving cognitive abilities [49]. Another study suggested that the SLAB51 probiotic formulation, containing eight live bacterial strains, improved impaired glucose metabolism in 3xTg-AD mice by reestablishing brain glucose transporters (GLUT1, GLUT3) and insulin-like growth factor receptor β (IGF1R), reducing phosphorylated tau aggregates, and inhibiting the accumulation of glycated hemoglobin and advanced glycation end products, thus delaying AD progression [50]. Meanwhile, the Lab4P probiotic consortia (containing *Bifidobacterium animalis* subsp. *lactis, Bifidobacterium bifidum*, *Lactobacillus acidophilus*, and *Lactobacillus plantarum*) demonstrated neuroprotective effects in AD mouse models [51]. Another study showed that a probiotic blend comprising *Levilactobacillus brevis*, *Bifidobacterium lactis*, and *Limosilactobacillus fermentum* relieved memory deficits, microglial activation, tau hyperphosphorylation, and Aβ deposition in 5xFAD mice [52]. However, in female *App^NL-G-F^* mice, supplementation with the probiotic VSL#3, a commercially available eight-strain lactic-acid bacterial blend, had negligible impact on brain Aβ, cytokine, or gliosis levels, while significantly reducing gut permeability and intestinal inflammation [46]. The limited brain effects may stem from the use of non-littermate wild-type mice as controls, low treatment duration/dose, the specific probiotic chosen, female-only mice, intervention initiated at an advanced amyloidosis/gliosis stage, and reliance on probiotic monotherapy without adjunct strategies such as exercise.

In conclusion, experimental studies have provided substantial evidence that probiotics, whether single or multi-strain, consistently reduced Aβ deposition, tau hyperphosphorylation, neuroinflammation, and synaptic damage across multiple AD models. These benefits are closely linked to gut microbiota restoration, underscoring the gut–brain axis as a promising therapeutic target for probiotic-based strategies against AD.

### 4.2. Clinical Trials

Few clinical trials have explored the potential benefits of probiotics in AD patients. For example, a 12-week multicenter, double-blind, placebo-controlled randomized clinical trial (RCT) with 90 AD patients in Iran found that supplementation with either *Lacticaseibacillus rhamnosus* HA-114 or *Bifidobacterium longum* R0175 markedly elevated the Mini-Mental State Examination (MMSE) total scores over time (*P*_Time×Group_ < 0.0001) [94]. Notably, *Bifidobacterium longum* R0175 yielded the most pronounced cognitive gains, with a mean increase of 4.86 points versus placebo (95% *CI*: 3.91–5.81; *p* < 0.0001), outperforming the *Lacticaseibacillus rhamnosus* group, which showed a smaller improvement of 4.06 points (95% *CI*: 3.11–5.01; *p* < 0.0001) [94]. The researchers further demonstrated that both probiotic interventions markedly attenuated serum inflammatory and oxidative stress markers (*P*_Time×Group_ < 0.0001), as well as enhanced quality of life and physical activity levels [95]. Moreover, a 12-week double-blind, active-controlled RCT with 32 AD patients conducted in the United States evaluated whether a higher dose of a five-strain probiotic blend (containing *Lactobacillus plantarum* PL-02, *Bifidobacterium animalis* subsp. *lactis* CP-9, *Bifidobacterium breve* Bv-889, *Bifidobacterium longum* subsp. *infantis* BLI-02, and *Bifidobacterium bifidum* VDD088) could modulate neurotrophic and inflammatory signatures [96]. Compared with baseline, the active arm showed serum BDNF rise from 7115.1 ± 4461.9 to 9678.5 ± 6652.9 pg/mL (*p* = 0.005), IL-1β fall from 2.7 ± 1.2 to 2.5 ± 1.2 pg/mL (*p* = 0.041), and superoxide dismutase (SOD) activity increase from 1.3 ± 0.3 to 1.6 ± 0.6 U/mL (*p* = 0.012) [96].

Several meta-analyses have offered valuable insights into the overall effects of probiotics on cognitive functions. Specifically, a meta-analysis of 12 RCTs involving 852 individuals with mild cognitive impairment (MCI) or AD revealed that probiotics significantly enhanced global cognitive function [99]. Similarly, another meta-analysis of RCTs indicated that probiotics significantly enhanced cognitive function in individuals with cognitive impairment or AD, with notable benefits from single-strain probiotics, longer treatment duration, and higher doses [100].

Together, these results suggested that probiotics may offer a safe and low-cost adjunct to standard care for AD, although head-to-head trials and longer-term evidence remain to be gathered before optimal strains, doses, and treatment lengths can be established.

## 5. Effects and Mechanisms of Prebiotics on Alzheimer’s Disease

Unlike probiotics which provide exogenous live bacteria, prebiotics serve as selective substrates for host microorganisms [11]. They have recently emerged as promising strategies for the prevention and management of AD. The effects and mechanisms of prebiotics on AD are summarized and shown in Figure 2 and Table 1 and Table 3 and are discussed in detail below.

### 5.1. Epidemiological Investigations

Epidemiological evidence supports the protective role of prebiotics against cognitive decline and AD. For example, in a longitudinal study of 1837 older adults living in northern Manhattan and free of dementia at enrollment (from Washington Heights-Inwood Columbia Aging Project), researchers found that every extra gram of daily fructan consumption was linked to a 24% lower risk of AD (95% *CI*: 0.60–0.97; *p* = 0.03), suggesting that higher fructan consumption may help protect against AD in older adults [101]. Furthermore, a study analyzing data from the National Health and Nutrition Examination Survey (NHANES) between 2011 and 2014 demonstrated that elderly American men taking nonfood pro- or prebiotic showed higher comprehensive cognitive function (*β* = 0.64, 95% *CI*: 0.08–1.19) and lower risk of cognitive impairment (*OR* = 0.08, 95% *CI*: 0.02–0.29) compared with those who did not consume pro- or prebiotic [102]. Similarly, another study using data from NHANES 2011–2014 revealed that individuals with cardiovascular disease history who consumed nondietary prebiotics had significantly higher z-scores in global cognition (*β* = 0.24, 95% *CI*: 0.03–0.46) and the CERAD-Delayed Recall Test (*β* = 0.35, 95% *CI*: 0.02–0.68) compared with those without prebiotic intake [103].

### 5.2. Experimental Studies

In experimental studies on AD models, carbohydrate-based prebiotics have been increasingly explored for their capacity to attenuate AD pathology and cognitive impairment. For instance, both lactulose and trehalose have been reported to reverse deficits in short-term memory and learning retrieval through attenuation of neuroinflammation and augmentation of autophagic signaling pathways in AD mice, with lactulose showing superior efficacy in enhancing synaptic protein expression levels [53]. Additionally, konjac glucomannan and oligo-glucomannan were shown to elevate spatial learning and memory in Aβ_1–42_-induced AD mice by activating the BDNF/PI3K/GSK3β pathway and increasing SCFAs production through the microbiota-SCFA-brain axis [54]. Meanwhile, mannan oligosaccharide (MOS) administration for 8 weeks significantly mitigated cognitive and behavioral disorders, as well as mental deficits in 5xFAD transgenic AD mice, partly due to gut microbiota modulation and increased SCFA production [55]. Unsaturated MOS derived from seaweed alginate curbed the aggregation of Aβ_1–42_ oligomer, dampened the expression of Aβ_1–42_, and decreased the concentrations of APP and β-secretase 1 in N2a-sw cells and primary cortex neurons from 3xTg-AD mice [56]. These effects were driven by its capacity to promote autophagy by inactivating the mechanistic target of rapamycin (mTOR) signaling pathway and boosting the fusion of autophagosomes and lysosomes [56]. Similarly, 6 weeks of fructo-oligosaccharide (FOS) administration in male APP/PS1 mice concurrently improved cognitive function and attenuated pathological alterations by reversing the altered microbial composition, increasing the level of glucagon-like peptide-1 (GLP-1), and decreasing the level of GLP-1 receptor (GLP-1R) [57]. FOS from *Morinda officinalis* also demonstrated therapeutic potential by restoring cognitive function in APP/PS1 mice by targeting the gut–brain axis [58]. This intervention displayed anti-inflammatory, antioxidant, and neuroprotective effects, including attenuation of neuronal apoptosis and brain tissue swelling [58]. On the other hand, galacto-oligosaccharides (GOS) demonstrated superior efficacy in mitigating cognitive decline in APP/PS1 mice compared with FOS and the FOS + GOS combination, primarily due to GOS’s capacity to adjust 5-HT and GABA levels by inhibiting the TLR4/MYD88/NF-κB pathway, and increasing *Lactobacillus* abundance in the gut microbiota [59]. Furthermore, κ-carrageenan oligosaccharides (KOS) effectively mitigated clinical manifestations of AD by downregulating levels of inflammatory markers and pro-inflammatory proteins in brain tissue in APP/PS1 mice [60]. Importantly, KOS restrained the overactivation of microglia, thereby reducing neuronal apoptosis and providing neuroprotection [60]. Additionally, another study found that chitooligosaccharide (COS) showed significant therapeutic potential by activating the nuclear factor erythroid 2-related factor 2 (Nrf2)/nuclear factor-κB (NF-κB) pathway [61]. Specifically, COS enhanced cognitive performance in APP/PS1 mice by elevating Nrf2 expression and decreasing Aβ accumulation along with NF-κB activation [61]. It also decreased systemic inflammatory mediators (TNF-α, IL-1β, IL-6) and key inflammatory markers (inducible nitric-oxide synthase/iNOS, cyclo-oxygenase 2/COX-2, NF-κB p65, NLRP3, caspase 1) in BV2 microglia stimulated by Aβ_25–35_ and lipopolysaccharides (LPS), while enhancing SK-N-SH cell viability [61]. Moreover, *dendrobium officinale* polysaccharides (DOP) effectively mitigated cognitive deficits, alleviated hippocampal neurodegeneration and Aβ plaque accumulation, and reinforced intestinal barrier function in AD mice [62]. Mechanistically, these benefits were attributed to DOP’s modulation of the gut microbiota, which involved reshaping its composition, restoring microbial diversity, normalizing disrupted metabolic profiles, and elevating SCFAs levels in AD mice [62].

Considering that the apolipoprotein ε4 allele (*APOE*4) is the predominant genetic risk factor for AD, emerging research has highlighted the promise of prebiotic inulin to curb AD progression through the brain-gut axis in *APOE*4 mouse models. For instance, a study found that inulin supplementation in *APOE*4 mice for 16 weeks alleviated gut dysbiosis, offering potential therapeutic effects for AD [63]. Notably, these benefits exhibited sex-specific differences: in female mice, inulin normalized gut microbiota α-diversity, reduced *Escherichia coli* more markedly, and dampened inflammatory responses, whereas in male mice, it reduced lactic acid bacteria more markedly and increased populations of SCFA-producing bacteria, primarily in acetate-producing bacteria [63]. Another study also indicated that in the *APOE*4 transgenic mouse model, dietary inulin reshaped the gut microbiota by enriching beneficial microbes and suppressing detrimental ones, mitigated neuroinflammation by downregulating inflammatory gene expression, and concurrently elevated systemic metabolism through increased levels of tryptophan metabolites, SCFAs, and other key metabolites [64].

Polyphenol-based compounds and other substrates that exhibit prebiotic-like effects have shown significant potential for AD. While these dietary compounds may not strictly meet the ISAPP criteria for selective utilization, they effectively modulate the gut microbiota to provide potential benefits. Notably, a study showed that isoorientin reduced AD-related markers in APP/PS1 mice, including lowering Aβ_42_ deposition, brain phosphorylated tau, and other inflammatory markers, while enhancing brain and serum IL-10 and IL-4 levels, by increasing microbial taxa in oral, cecal, and fecal samples [65]. Besides, quercetin-3-O-glucuronide demonstrated potential benefits in AD by alleviating neuroinflammation and brain insulin resistance, reducing Aβ accumulation and tau hyperphosphorylation, normalizing cAMP response element binding protein (CREB)/BDNF levels, reversing cognitive deficits, and rebalancing SCFAs levels and gut microbiota in Aβ_1–42_-induced AD-like mice and SH-SY5Y cells [66]. In addition, curcumin might enhance spatial learning and memory and reduce amyloid-plaque aggregation in APP/PS1 mice by modulating the abundance of bacterial taxa and generating eight metabolites through gut microbiota transformation [67]. Furthermore, in a D-galactose/AlCl_3_-induced AD mouse model, supplementation with resveratrol-selenium (Se)-peptide nanocomposites boosted cognitive performance by reducing Aβ clustering and burden, alleviating Aβ-induced oxidative damage and neuroinflammation via key signaling pathways, and rectifying gut microbiota imbalance, especially among taxa involved in oxidative stress and inflammatory responses, thus indicating potential to decelerate AD progression [68]. Additionally, the prebiotic R13, a prodrug of the BDNF-mimetic 7,8-DHF, mitigated AD in 5xFAD mice by reducing amyloid deposits and promoting the growth of probiotic *Lactobacillus salivarius* [69]. Both R13 and *Lactobacillus salivarius* curbed the CCAAT/enhancer binding protein β/asparagine endopeptidase (C/EBPβ/AEP) axis, effectively reducing oxidative stress and intestinal permeability [69]. Meanwhile, in vitro fermentation using feces from APP/PS1 mice revealed that Se-enriched proteins, particularly Se-enriched soybean protein (H-SBP) and Se-enriched cardamine violifolia protein (H-CVP), demonstrated prebiotic-like effects by modulating gut microbiota [70]. H-CVP promoted the proliferation of *Bacteroidetes* strains, whereas H-SBP markedly elevated *Firmicutes* and *Lactobacillaceae* levels, thereby alleviating intestinal inflammation and cognitive impairment in APP/PS1 mice [70].

In conclusion, prebiotics consistently improved cognition and curbed Alzheimer-like pathologies in experimental models by modulating gut microbiota, dampening neuroinflammation, and enhancing autophagy. Additional benefits included anti-inflammatory and neuroprotective effects, with some compounds targeting genetic risk factors such as *APOE*4. Although these mechanistic findings are encouraging, well-designed clinical studies are needed to translate prebiotic interventions into practical strategies for AD prevention and management in the future.

## 6. Effects and Mechanisms of Synbiotics on Alzheimer’s Disease

Synbiotics represent an integration of the live microorganisms and selective substrates discussed in the previous sections [12]. Synbiotics have shown potential benefits in ameliorating AD pathology and improving cognitive function in various experimental models, with some evidence from initial clinical trials (Figure 2 and Table 1 and Table 2).

### 6.1. Experimental Studies

Experimental studies have revealed the neuroprotective potential of synbiotics in the *Drosophila* model of AD. For example, a novel synbiotic formulation, comprising *Lactobacillus fermentum*, *Lactobacillus plantarum*, *Bifidobacterium infantis* and a novel polyphenol-rich prebiotic, reversed Aβ accumulation and acetylcholinesterase activity and improved survival and locomotion in a transgenic humanized *Drosophila melanogaster* model of AD [71]. These effects likely involved gut–brain-axis pathways related to immune signaling and metabolic stability, mitochondrial dysfunction, and oxidative stress, potentially mediated by peroxisome proliferator-activated receptor gamma (PPARγ), thereby exerting protective effects against AD progression [71].

Synbiotics have shown significant benefits in AD in rodent models. In particular, a study found that synbiotic treatment with *Clostridium sporogenes* and xylan in the 5xFAD mouse model improved cognitive deficits, reduced brain Aβ levels, restored synaptic structure, dampened neuroinflammation, shifted gut microbiota toward beneficial bacteria, and maintained gut barrier integrity [72]. Elevated indole-3-propionic acid synthesis by the gut microbiota could underlie these beneficial effects, highlighting its potential as a promising microbiota-directed strategy for ameliorating AD [72]. Meanwhile, in the APP transgenic mouse model of AD, a synbiotic containing six probiotics (*Bacillus coagulans*, *Bacillus natto*, *Bifidobacterium longum*, *Bifidobacterium breve*, *Lactobacillus casei*, and *Lactobacillus acidophilus*) and a prebiotic (inulin) regulated the gut–brain axis, thereby alleviating AD-like deficits, which included lowering Aβ_42_ levels, mitigating the inflammatory response, promoting neurogenesis in the hippocampus, and alleviating cognitive impairment [73]. In addition, synbiotics (*Bifidobacterium lactobacillus*, *Lactobacillus acidophilus*, and xylo-oligosaccharide) administered over 3 months in APP/PS1 mice significantly enhanced learning and memory by stimulating PPARs signaling pathways, which concurrently modulated the gut microbiota and delayed AD progression [74]. Moreover, a study highlighted that NMN synbiotics, encompassing lactulose, *Lactobacillus plantarum*, and β-nicotinamide mononucleotide, profoundly affected the structure of the metabolic profiles and the gut microbiota in APP/PS1 mice [75]. Specifically, this intervention reconfigured the gut microbiota and fine-tuned pivotal metabolic pathways, thereby diminishing Aβ-induced amyloid plaques in AD mice [75]. Furthermore, intervention with prebiotics (a plant and fiber extract mixture, including inulin and FOS) together with probiotics (*Lactobacillus rhamnosus* and *Lactobacillus paracasei*) in APP/PS1 mice reduced hippocampal CA3 Aβ plaques, preserved CA1 neurons, and modulated astrocyte activation and microglial reactivity across both CA regions [76]. Meanwhile, in a rat model of preclinical AD, ProBiotic-4, containing *Lactobacillus casei*, *Lactobacillus acidophilus*, *Bifidobacterium lactis*, and *Bifidobacterium bifidum*, mixed with prebiotic FOS, effectively alleviated pretangle tau pathology, as demonstrated by improved spatial learning, decreased inflammation, as indicated by reduced ionized calcium-binding adapter molecule 1 (Iba1) and cluster of differentiation 68 (CD68) expression, and inhibition of GSK-3β in female rats, all through the gut–brain axis [77]. However, synbiotic (containing eight strains of lactic acid-producing bacteria and oligofructose-enriched-inulin) plus antibiotic treatment in the *App^NL-G-F^* AD mouse model showed minimal benefits on cognitive abilities in mice of both sexes [78]. The researchers of the study hypothesized that, in males, specific bacteria may be critical for plaque deposition [78]. The depletion of these microbes by antibiotics might facilitate plaque clearance, whereas subsequent synbiotic supplementation may return the gut to a state of disease-promoting dysbiosis [78].

Overall, experimental studies in animal models (such as *Drosophila*, mice, and rats) have suggested that synbiotics positively affect AD. They improved cognitive function and AD pathology by modulating gut microbiota, decreasing Aβ accumulation, curbing neuroinflammation, and influencing various gut–brain axis pathways.

### 6.2. Clinical Trials

Several clinical trials have reported mixed results regarding the effects of synbiotics in AD. For instance, in an uncontrolled clinical trial with 13 AD patients in Brazil, a 90-day intake of kefir-based synbiotic promoted notable enhancements in cognitive performance, such as improved memory retention, better visual-spatial and abstract thinking abilities, and stronger executive and language functions, by modulating inflammatory response, oxidative burden, and blood cell injury [97]. This suggested its potential as a complementary approach to delay AD progression. However, in another randomized, placebo-controlled, double-blind RCT of 60 patients with mild to moderate AD in Iran, a 12-week synbiotic supplementation with a blend of seven bacterial strains (*Streptococcus thermophilus*, *Bifidobacterium breve*, *Bifidobacterium longum*, *Lactobacillus acidophilus*, *Lactobacillus bulgaricus*, *Lactobacillus casei*, and *Lactobacillus rhamnosus*) as probiotics and a prebiotic (FOS) failed to boost cognitive or physical capabilities in elderly subjects [98]. This outcome might have been affected by the limited sample size, heterogeneity of probiotic strains and dosages employed across studies. In conclusion, these inconsistent findings highlighted the need for further research to elucidate the therapeutic potential of synbiotics in AD patients. Future research should prioritize validated cognitive scales and functional outcomes to ensure that the observed changes are clinically meaningful for patients.

## 7. Effects and Mechanisms of Postbiotics on Alzheimer’s Disease

Postbiotics, representing a novel class of therapeutic candidates, could directly or indirectly influence AD progression through diverse neuroprotective and anti-inflammatory mechanisms (Figure 2 and Table 2 and Table 3).

### 7.1. Epidemiological Investigations

As novel therapeutic candidates, postbiotics have shown potential in influencing the progression of AD, and several epidemiological studies have uncovered intriguing links between postbiotics and AD. For example, in a cross-sectional study in China involving 19 healthy controls and 19 AD patients with positive amyloid positron emission tomography scans, serum and fecal propionic acid levels were markedly lower in AD patients (*p* < 0.0002) [81]. Another study in China with 29 AD patients and 29 age-matched healthy controls found that AD patients had markedly lower lysophosphatidylcholine (LPC) concentrations in their serum (*p* < 0.05), with similar reductions observed in fecal samples [86]. These findings from epidemiological investigations collectively implicated postbiotics as potentially influential factors in AD pathology.

### 7.2. Experimental Studies

Experimental studies have further explored the mechanisms by which postbiotics may affect AD pathology. The influence of gut microbiota-derived metabolites, particularly SCFAs, on AD is a rapidly evolving area of research. Specifically, one study used cultured human THP-1 monocytic cells and differentiated human HL-60 myelomonocytic cells as models, exposing them individually or in combination to formate, acetate, propionate, butyrate, and valerate [79]. The results revealed that SCFAs markedly lowered the secretion of key inflammatory mediators, including TNF-α, IL-1β, cytotoxins, and monocyte chemoattractant protein (MCP-1), in immune-stimulated THP-1 cells [79]. Additionally, valerate and formate both decreased the phagocytic activity of THP-1 cells, while formate suppressed the respiratory burst triggered by N-formylmethionine-leucyl-phenylalanine (fMLP) in HL-60 cells, leading to a reduction in ROS generation [79]. These results indicated that SCFAs have the potential to modulate specific microglial functions that are impaired in AD [79]. In another study, acetate was administered to a male APP/PS1 mouse model for 4 weeks, which significantly mitigated the cognitive deficits and reduced the CD11b level [80]. It also suppressed JNK, ERK, and NF-κB p65 phosphorylation, reduced IL-1β and COX-2 levels, and upregulated G-protein-coupled receptor 41 (GPR41) in Aβ-stimulated BV2 cells [80]. Similarly, propionic acid, derived from *Akkermansia muciniphila*, demonstrated efficacy in AD mouse models and cultured hippocampal neuronal cells by modulating mitochondrial homeostasis through downregulation of mitochondrial fission protein (DRP1) via GPR41 and enhancement of PTEN-induced kinase 1 (PINK1)/Parkin RBR E3 ubiquitin protein ligase (Parkin)-mediated mitophagy via GPR43 [81]. This intervention preserved mitochondrial function, thereby ameliorating cognitive impairment and mitigating AD progression [81]. Moreover, in a nine-month dietary intervention in APP/PS1 mice, researchers suggested that SCFAs could modulate gut microbiota homeostasis, diminish Aβ plaques and tau phosphorylation, and augment astrocyte-neuron signaling via the glutamate-glutamine cycle, thereby collectively alleviating cognitive deficits and slowing AD progression [82]. However, not all studies support a protective role: microbiota-derived SCFAs have been reported to increase Aβ plaque burden and boost microglial convergence to plaques, while simultaneously diminishing intracellular Aβ levels in microglia in germ-free APP/PS1 mice [83]. The different SCFAs’ effects might be associated with the specific disease conditions and dose/duration of treatment.

In addition to SCFAs, other metabolites of the gut microbiota, such as tryptophan metabolites, LPC, and phenolic compounds, have also been implicated in AD. Notably, gut microbiota-derived tryptophan metabolites, specifically indoles, such as indole, indole-3-carboxyaldehyde (Icld), indole-3-acetic acid (IAA), indole-3-propionic acid (IPA), and indole-3-lactic acid (ILA), have shown neuroprotective effects in HT-22 cells [84]. These metabolites activated the GPR30/AMP-activated protein kinase (AMPK)/silent information regulator 1 (SIRT1) axis in vitro [84]. Further validation in D-galactose-induced aging mice demonstrated that their neuroprotective effects were mediated through the GPR30/AMPK/SIRT1 pathway, suggesting their potential to delay the progression of AD and related disorders [84]. Likewise, a study found that 4 weeks of indole treatment in male APP/PS1 mice could promote cognitive function, reduce Aβ and hyperphosphorylated tau levels, strengthen gut barrier integrity, enhance synaptic plasticity, and alleviate neuroinflammation and inflammatory cytokine (IL-1β, IL-6, IL-18, and TNF-α) release [85]. These benefits were attributed to the modulation of the aryl hydrocarbon receptor pathway and inhibition of NLRP3 inflammasome formation [85]. Moreover, in the 5xFAD mouse model, administration of LPC from *Bacteroides ovatus* markedly diminished Aβ accumulation, restored synaptic function, reduced gliosis, mitigated myelin degeneration and enhanced cognitive function by engaging the orphan receptor GPR119 to curb acyl-CoA synthetase long-chain family member 4 (ACSL4) expression, thus inhibiting ferroptosis and modulating AD [86]. Additionally, flavonoid-derived phenyl-γ-valerolactone metabolites, major products of gut microbial metabolism of flavonoids, have been shown to mitigate β-oligomer-induced cytotoxicity in yeast and mammalian cells, improve memory deficits, and attenuate neuroinflammation in an acute mouse model of Aβ oligomer (oAβ)-induced neurotoxicity, which is relevant to AD pathology [87].

Exopolysaccharides (EPSs) and extracellular vesicles (EVs), which are bioactive substances secreted by microbes, belong to the category of postbiotics and have been shown to affect AD. For example, EPSs from lactic acid bacteria protected human neuroblastoma SH-SY5Y cells from Aβ-mediated neurotoxicity by preserving antioxidant status and enzyme activities (SOD, catalase/CAT, and glutathione peroxidase/GPx) and activating ERK1, ERK2, JUN (Jun proto-oncogene, AP-1 transcription factor subunit), JNK, NF-κB p65, and p38 while inhibiting AKT [88]. This action offered the potential to mitigate AD driven by oxidative stress [88]. Additionally, *Lactobacillus paracasei*-derived EVs could mitigate AD pathology and memory loss in APP/PS1 mice via upregulating SIRT1 and methyl-CpG binding protein 2 (MeCP2) [89]. In HT22 neurons challenged with Aβ, the vesicles rescued the expression of neurotrophins (*neurotrophin 3*/*Nt3*, *Nt4/5*, *Bdnf*) and *tropomyosin receptor kinase B* (*TrkB*), while simultaneously rescuing the amyloid-degrading enzymes including *neprilysin* (*Nep*), *matrix metalloproteinase 2* (*Mmp-2*), and *Mmp-9* [89].

Other postbiotics have also been explored for their potential to influence AD progression. For example, a study investigated the benefits of postbiotics derived from three lactic acid bacteria strains (*Levilactobacillus brevis* CRL 2013, *Lactobacillus delbrueckii* subsp. *lactis* CRL 581, and *Enterococcus mundtii* CRL 35) in the microglia cell line BV-2 [90]. These postbiotics could reduce oxidative stress induced by oAβ_1–42_, lower the expression of inflammatory cytokines (TNF-α, IL-6, and IL-1β), and inhibit acetylcholinesterase (AChE) activity [90]. In addition, a study found that postbiotic treatment containing tyndallized *Lactobacillus acidophilus* and *Bifidobacterium longum* lysates could disaggregate Aβ_1–40_ aggregates through the chelation of Zn^2+^ and Cu^2+^ ions in APP/PS1 mice [91]. The same treatment also reduced the expression of the mouse APP gene and endogenous human APP transgenic protein and enhanced mitochondrial ATP-dependent Lon protease homolog 1 (LONP1) activity [91]. Meanwhile, in a polymicrobial mouse model of periodontal disease, nisin, a *Lactococcus lactis*-derived probiotic bacteriocin, alleviated brain microbiome dysbiosis, diminished neuroinflammation (reductions in IL-1β, TNF-α, and IL-6), and exerted positive impacts on AD-like pathogenic changes by markedly lowering phosphorylated tau deposition, total tau, and Aβ_42_ [92]. Furthermore, a study demonstrated that APP/PS1 mice, when administered heat-inactivated *Streptococcus thermophilus* MN-ZLW-002 over a three-month period, exhibited notable alleviation of cognitive impairment, especially in spatial memory, by virtue of elevated colonic propionic acid concentrations and augmented antioxidant defenses in the hippocampus, all facilitated through the gut–brain axis [93].

In summary, postbiotics, encompassing a range of bioactive substances such as SCFAs, tryptophan metabolites, EPSs, and EVs, have shown diverse effects on AD. Many studies highlighted their potential neuroprotective and anti-inflammatory benefits, but several others suggested variable outcomes, with some postbiotics having no impact. Overall, postbiotics present a promising yet complex therapeutic avenue for AD, warranting further investigation to fully understand their mechanisms and optimize their potential benefits in humans, with special attention to adverse effects.

## 8. Conclusions and Perspectives

This narrative review suggests the profound and multifaceted involvement of the gut–brain axis in the pathogenesis of AD. The current evidence can be graded into three levels, whereby preclinical animal models provide robust mechanistic data while epidemiological and clinical findings remain preliminary. Emerging evidence from epidemiological, experimental, and clinical studies indicates a potential for targeting gut microbiota through PPSPs. It is essential to distinguish between these robust preclinical findings and preliminary clinical data. These PPSPs exert their beneficial effects via diverse mechanisms. Probiotics, both single and multi-strain, have shown potential in mitigating AD and improving cognitive function by rebalancing gut microbiota, reducing Aβ plaques and tau phosphorylation, and modulating inflammatory responses. Similarly, prebiotics and dietary compounds with prebiotic-like effects, such as polyphenols, have shown promise in improving cognitive function and decreasing AD risk, often by fostering beneficial microbial growth and systemic metabolic improvements. Synbiotics have also exhibited the capacity to alleviate AD-like deficits and enhance learning and memory. Finally, postbiotics, encompassing microbial metabolites such as tryptophan derivatives and SCFAs, alongside microbial components like EPSs and EVs, are increasingly recognized for their direct neuroprotective and anti-inflammatory properties, offering a novel frontier in AD management.

The observed variability in clinical outcomes is often attributable to factors such as strain specificity and dosage. Differences in intervention duration and the disease stage of participants also significantly influence the results. However, most mechanistic data currently stem from rodent models and in vitro studies, with limited validation in humans. This translation gap remains a major challenge because animal models cannot fully replicate the complex and decades-long progression of human AD. Current clinical evidence is limited by small sample sizes, significant study heterogeneity, and short intervention duration. Furthermore, confounding variables such as baseline diet, medications, and individual microbiota profiles may influence outcomes. Future research should prioritize in-depth human mechanistic studies. These studies should profile gut microbiota and metabolite changes in PPSP-treated AD patients. Crucially, these alterations should be linked to brain imaging or CSF biomarkers of AD pathology, such as Aβ and tau levels.

PPSPs should currently be viewed as a promising adjunctive or complementary strategy rather than an established primary treatment for AD. Transitioning from preliminary evidence to established AD therapy will require large-scale trials that prioritize clinically meaningful endpoints, such as cognitive performance and functional recovery, over mechanistic markers alone. Future research must also prioritize safety and quality control for the real-world implementation of PPSPs. This includes ensuring accurate strain identification and maintaining stable PPSP microbial counts until the end of shelf life. Preventing contamination and verifying the defined composition and dosage of PPSP preparations are also essential. These factors are especially critical for older, frail, or immunocompromised AD patients who may be more vulnerable to adverse effects. Addressing these regulatory and standardization challenges will determine the scalability and long-term success of PPSPs in clinical AD management.

## Figures and Tables

**Table 1:** The effects and mechanisms of probiotics, prebiotics, synbiotics, and postbiotics on Alzheimer’s disease from experimental studies.

Study Type	Model	Intervention	Dose	Duration	Primary Endpoints	Main Mechanisms	Ref.
**Probiotics**							
In vivo	D-galactose/AlCl_3_-induced Wistar male rats	*Lactobacillus plantarum* MA2	1 × 10^8^ CFU/kg/day or 1 × 10^9^ CFU/kg/day	13 weeks	Alleviated AD progression	↓ neuronal damage, Aβ plaque deposition, and amyloid protein-induced cytotoxicity ↓ cognitive impairment and anxiety-like behaviors ↓ microglial activation and neuroinflammation via the TLR4/MYD88/NLRP3 signaling pathway Regulated the gut microbiota and glycometabolism	[31]
In vivo	D-galactose/AlCl_3_-induced ICR mice	*Lactobacillus plantarum* DP189	10 mL/kg/day (1 × 10^9^ CFU/mL)	10 weeks	Ameliorated cognitive deficits and AD pathological changes	↑ 5-HT, GABA, and dopamine ↓ neuronal damage, Aβ deposition, and tau pathology Regulated the gut microbiota and PI3K/AKT/GSK-3β pathway	[32]
In vivo	APP/PS1 mice	Encapsulated *Lactiplantibacillus plantarum*	1 × 10^8^ CFU/kg/day	6 weeks	Improved AD symptoms	↓ brain neuroinflammation and neuronal damage ↓ Aβ deposition and tau protein phosphorylation ↓ intestinal barrier damage ↑ PSD-95 and synaptic plasticity ↑ SCFAs and restore intestinal microbiota composition	[33]
In vivo	Choline-treated male APP/PS1 mice	Memantine + *Lactobacillus plantarum*	1 mg/mL/day + 1 × 109 CFU/mL/day	12 weeks	Attenuated cognitive impairment	↓ Aβ levels in the hippocampus ↑ neuronal integrity and plasticity ↓ TMAO synthesis, neuroinflammation, and clusterin levels Remodeled intestinal microbiota	[34]
In vivo	2-month-old C57BL/6J mice	*Bifidobacterium breve* MCC1274	1 × 10^9^ CFU/day in 200 μL saline	5 times a week for 4 months	Attenuated AD-related pathologies	↓ soluble hippocampal Aβ_42_ and PS1 protein ↓ phosphorylated tau levels and neuroinflammation ↑ AKT/GSK-3β pathway ↓ microglial activation and ↑ synaptic protein	[35]
In vivo	17-month-old *App^NL-G-F^* mice	*Bifidobacterium breve* MCC1274	1 × 10^9^ CFU/day	5 times a week for 4 months	Attenuated AD-related pathologies	↓ phosphorylated ERK1/2, JNK and HSP90 ↓ chronic stress and tau hyperphosphorylation ↑ hippocampal synaptic protein levels and ↓ neuronal activity	[36]
In vivo	Aβ_1–42_-treated male C57BL/6J mice	*Bifidobacterium breve* CCFM1025 or *Bifidobacterium breve* WX	200 μL/day (3 × 10^9^ CFU/mL)	6 weeks	Improved brain function	↑ synaptic plasticity ↑ BDNF, FNDC5, and PSD-95 Modulated gut microbiota composition	[37]
In vivo	Aβ-injected C57BL/6J mice	*Bifidobacterium breve* CCFM1025	200 μL (5 × 10^9^ CFU/mL)	6 weeks	Reversed the metabolite profile disrupted by Aβ-injection	Altered metabolites mainly involved amino acid metabolism	[38]
In vivo	APP/PS1 mice	*Bifidobacterium breve* HNXY26M4	1 × 10^9^ CFU/day	12 weeks	Attenuated cognitive deficits and neuroinflammation	↓ neuroinflammation and synaptic dysfunction ↓ brain oxidative damage ↑ function of BBB and intestinal barrier Restored the composition of gut microbiota and SCFAs	[39]
In vivo	APP/PS1 mice	*Bifidobacterium longum* 1714	0.2 mL/10 g BW/day (1 × 10^9^ CFU/mL)	6 months	Alleviated the pathological changes of AD	↓ Aβ deposition ↓ microglial activation ↓ IL-1β, IL-6, TNF-α, and IFN-γ	[40]
In vivo	4-month-old APP/PS1 mice	*Bifidobacterium lactis* Probio-M8	0.2 mL/10 g BW/day (1 × 10^9^ CFU/mL)	45 days	Alleviated AD pathophysiology	↓ Aβ plaque burden ↓ gut microbiota dysbiosis ↓ cognitive impairment	[41]
In vivo	Intracerebroventricularly Aβ-injected C57BL/6 male mice	*Bifidobacterium lactis* CBT BL3	100 μL/day (2 × 109 CFU/mL)	6 weeks	Mitigated memory impairment	↓ the expression of apoptosis-related proteins such as caspase-9, caspase-3 ↓ abnormal over-phosphorylation of MAPKs such as ERK1/2, p38 MAPK, and JNK in the brain tissue	[42]
In vivo	High-fat diet-fed APP/PS1 mice	*Akkermansia muciniphila* GP01	5 × 10^9^ CFU/day	6 months	Alleviated cognitive deficits and amyloid pathology	↓ Aβ_40–42_ levels in the cerebral cortex ↓ the fasting blood glucose and serum diamine oxidase levels ↓ blood lipid levels ↓ hepatic steatosis and scapular brown fat whitening ↓ intestinal barrier dysfunction Attenuated the reduction of colonic mucus cells	[43]
In vivo	APP/PS1 mice	*Clostridium butyricum* WZMC1016	200 µL/day (1 × 10^9^ CFU/mL)	4 weeks	Reduced microglia-driven neuroinflammation in AD	↓ cognitive impairment ↓ Aβ deposits, microglia activation, and neurodegeneration ↓ TNF-α and IL-1β Reversed abnormal gut microbiota and butyrate	[44]
In vivo	6-month-old male APP/PS1 mice	*Saccharomyces boulardii*	0.2 mL/day (5 × 108 CFU/mL)	4 weeks	Mitigated cognitive deficits	↓ microglia activation and the TLRs pathway ↓ dysbiosis, neuroinflammation and synaptic injury	[45]
In vivo	*App^NL-G-F^* mice	VSL#3 (a commercially available probiotic cocktail of eight strains of lactic acid-producing bacteria: *Lactobacillus plantarum*, *Lactobacillus delbrueckii* subsp. *Bulgaricus*, *Lactobacillus paracasei*, *Lactobacillus acidophilus*, *Bifidobacterium breve*, *Bifidobacterium longum*, *Bifidobacterium infantis*, and *Streptococcus salivarius* subsp. *Thermophilus*)	5 mL/25 g BW/day (0.32 × 10^9^ CFU/25 g BW)	8 weeks	Ameliorated intestinal pathophysiology in a mouse model of AD	↓ IL-1β, TNF-α, and LCN-2 ↓ intestinal inflammation and gut permeability with minimal effect on Aβ, cytokine, or gliosis levels	[46]
In vivo	6-month-old SAMP8 mice	Probiotic-2 (containing *Bifidobacterium lactis* and *Lactobacillus rhamnosus*) or probiotic-3 (comprising *Bifidobacterium lactis*, *Lactobacillus acidophilus*, and *Lactobacillus rhamnosus*)	1 × 10^9^ CFU/day	8 weeks	Ameliorated Alzheimer’s-like cognitive impairment and pathological changes	↓ neural injury, Aβ and tau pathology and neuroinflammation Regulated the phosphorylation of the AKT/GSK-3β pathway	[47]
In vivo	APP/PS1 mice	*Lactobacillus* strains (*Lactobacillus paracasei* D3.5, *Lactobacillus rhamnosus* D4.4, *Lactobacillus plantarum* D6.2, *Lactobacillus rhamnosus* D7.5 and *Lactobacillus plantarum* D13.4) and *enterococcus* strains (*Enterococcus raffinosus* D24.1, *Enterococcus* INBio D24.2, *Enterococcus avium* D25.1, *Enterococcus avium* D25.2 and *Enterococcus avium* D26.1)	1 × 10^11^ CFU/day	16 weeks	Mitigated AD pathology and cognitive decline	↓ cognitive decline ↓ Aβ aggregation, microglia activation, and neuroinflammation ↓ inflammatory microbiome ↓ gut permeability and inflammation in both systemic circulation and the brain ↑ BBB tight junctions via higher expression of Zo-1 and Claudin	[48]
In vivo	3xTg-AD and 3xTg; Thy1-YFP transgenic male mice	BIOCG formulation, containing *Lactobacillus plantarum* (Lp3a; 50%), *Bifidobacterium animalis* subsp. *lactis* (Bla019; 25%), and *Bifidobacterium longum* (BL5b; 25%)	1 × 10^9^ CFU/kg/day	3 months	Protected against Alzheimer’s-related cognitive deficits	↑ microbial diversity ↑ dendritic spine maturation ↑ cognitive function ↓ Aβ burden and neuroinflammation	[49]
In vivo	8-week-old male 3xTg-AD mice	SLAB51 probiotic formulation, containing eight different live bacterial strains: *Streptococcus thermophilus* DSM 32245, *Bifidobacterium lactis* DSM 32246, *Bifidobacterium lactis* DSM 32247, *Lactobacillus acidophilus* DSM 32241, *Lactobacillus helveticus* DSM 32242, *Lactobacillus paracasei* DSM 32243, *Lactobacillus plantarum* DSM 32244, and *Lactobacillus brevis* DSM 27961	2 × 10^11^ bacteria/kg/day	48 weeks	Restored glucose homeostasis in a mouse model of AD	↑ key glucose transporters (GLUT3, GLUT1) and IGF1R ↓ phosphorylation of AMPK and AKT ↓ phosphorylated tau aggregates ↓ the time-dependent increase of glycated hemoglobin and the accumulation of advanced glycation end products	[50]
In vivo; In vitro	3xTg-AD mice; high-fat diet-fed 3xTg-AD mice; Human SH-SY5Y neuronal cell	Lab4P probiotic consortium comprising *Lactobacillus acidophilus* CUL21, *Lactobacillus acidophilus* CUL60, *Lactobacillus plantarum* CUL66, *Bifidobacterium bifidum* CUL20 and *Bifidobacterium animalis* subsp. *lactis* CUL34	5 × 108 CFU/day; 1 × 109 CFU/mL	24 weeks; 12 weeks; N/A	Exerted cognitive neuroprotective effects	↓ mRNA levels of the pro-inflammatory cytokine, IL-6 Prevented disease-associated deteriorations in NOR, hippocampal neuron spine density (particularly thin spines) and mRNA expression in hippocampal tissue Protected undifferentiated SH-SY5Y cells against rotenone, serum deprivation and D-galactose	[51]
In vivo	4-month-old 5xFAD mice	*Bifidobacterium lactis* KL101, *Limosilactobacillus fermentum* KL271, and *Levilactobacillus brevis* KL251	8 × 10^7^ CFU/day	3 months	Reduced AD pathological features	↓ microglial activation ↓ tau hyperphosphorylation ↓ Aβ deposition	[52]
**Prebiotics**							
In vivo; In vitro	Aβ_23–35_-injected C57BL/6J mice; mouse primary hippocampal neurons	Lactulose or trehalose	200 mg/kg, 0.1 mL; different concentrations	28 days; 48 h	Ameliorated short-term memory and learning retrieval deficits	↓ neuroinflammation ↑ autophagic signaling pathways Modulated gut microbiome and insulin sensitivity	[53]
In vivo	Aβ_1–42_ induced male C57BL/6J mice (8–10 weeks old)	Konjac glucomannan or oligo-glucomannan	800 mg/kg	12 weeks	Increased the spatial learning and memory	↓ Aβ accumulation and tau hyperphosphorylation ↑ *Bdnf*, *Trkb*, *Pi3k* and *Akt* ↓ *Gsk3β* ↑ bacteria belonging to *Alistipes* and SCFAs	[54]
In vivo	6-month-old male 5xFAD mice	Mannan oligosaccharide	0.12% *w*/*v*	8 weeks	Mitigated the cognitive and behavioral disorders as well as mental deficits	Balanced HPA-axis and redox status ↓ corticosterone and corticotropin-releasing hormone ↑ norepinephrine expression ↓ Aβ accumulation and amygdala of the brain ↓ neuroinflammatory responses ↓ gut barrier integrity damage and the LPS leak ↑ *Lactobacillus* and ↓ *Helicobacter* ↑ butyrate formation and related microbial levels	[55]
In vitro	N2a-sw cells and primary cortex neurons from 3xTg-AD mice	Unsaturated mannuronate oligosaccharide	1 mg/mL	24 h	Ameliorated Aβ pathology	↓ aggregation of oAβ_1–42_ and expression of Aβ_1–42_ ↓ APP and BACE1 ↑ autophagy (inactivation of mTOR signaling pathway and the facilitation of the fusion of autophagosomes and lysosomes)	[56]
In vivo	6-month-old male APP/PS1 mice	Fructo-oligosaccharides	2% *w*/*w*	6 weeks	Ameliorated cognitive deficits and pathological changes	Reversed the altered microbial composition ↑ synapsin I and PSD-95 ↓ activation of JNK ↑ GLP-1 and ↓ GLP-1R ↓ cognitive deficits and Aβ deposition	[57]
In vivo	2-month-old APP/PS1 mice	Oligosaccharides from *Morinda officinalis*	50 mg/kg/day or 100 mg/kg/day	6 months	Enhanced learning and memory abilities	Regulated gut microbiota ↓ oxidative stress and inflammation disorder ↓ swelling of brain tissues, neuronal apoptosis ↓ tau and Aβ_1–42_	[58]
In vivo	6-month-old male APP/PS1 mice	Fructo-oligosaccharides and galacto-oligosaccharides	0.4 g/day (used independently), 0.04 g/day + 0.36 g/day (used in combination)	6 weeks	Modulated the microbiota–gut–brain axis and ameliorated cognitive impairment	↓ Aβ burden and pro-inflammatory IL-1β and IL-6 levels ↓ proteins of the TLR4-MYD88-NF-κB pathway in the colons and cortexes ↓ GABA and ↑ 5-HT ↑ *Lactobacillus* and *Bifidobacterium*	[59]
In vivo; In vitro	APP/PS1 mice; Murine microglia line N9 and mouse neuroblastoma Neuro-2a cells (LPS-exposed)	κ-carrageenan oligosaccharides	100 mg/kg; 100 μg/mL	3 times a week for 4 weeks; 6 h	Mitigated clinical manifestations of AD	↓ inflammatory markers and pro-inflammatory proteins in brain tissue ↓ overactivation of microglia ↓ neuronal apoptosis ↓ APP and Aβ_1–42_ deposition ↓ iNOS, NF-L, Tau, and ApoE ↑ CSP-α	[60]
In vivo; In vitro	Male APP/PS1 mice; mouse (Aβ_25–35_ + LPS)-exposed BV2 microglia microglial cells; human neuroblastoma SK-N-SH cells	Chitooligosaccharide	200 mg/kg/day; 200 μg/mL; different concentrations of COS	16 weeks; 4 h or 24 h; 4 h	Ameliorated cognitive deficits and neuroinflammation	↑ Nrf2 and HO-1 ↓ Aβ accumulation and NF-κB activation ↓ IL-6, IL-1β, TNF-α ↓ iNOS, COX-2, NLRP3, caspase 1, NF-κB p65	[61]
In vivo	6-month-old male APP/PS1 mice	*Dendrobium officinale* polysaccharides	400 mg/kg/day	6 weeks	Improved cognitive impairment and protected the nerves	Modulated gut microbiota ↓ hippocampal neuronal damage and Aβ plaque deposition ↑ intestinal barrier integrity and microbial diversity ↑ SCFAs	[62]
In vivo	C57BL/6 mouse model with human-targeted replacement *APOE* (*ε4* in the *E4*FAD line and *ε3* in the *E3*FAD line)	Inulin	8% in diets	16 weeks	Enhanced gut microbial metabolism and reduced inflammation with sex-specific implications	In females: ↓ *Escherichia coli* and inflammation-associated pathway responses In males: ↑ SCFA-producing bacteria (related to acetate)	[63]
In vivo	C57BL/6 mouse model with human-targeted replacement *APOE* (*ε4* in the *E4*FAD line and *ε3* in the *E3*FAD line)	Inulin	8% in diets	16 weeks	Enhanced systemic metabolism and reduced neuroinflammation	↑ beneficial microbiota and ↓ harmful microbiota ↑ metabolism in the cecum, periphery and brain ↑ SCFAs, tryptophan-derived metabolites, bile acids, glycolytic metabolites and scyllo-inositol ↓ inflammatory gene expression	[64]
In vivo	Male APP/PS1 mice	Isoorientin	25 or 50 mg/kg	60 days	Impacted AD markers	↓ brain phospho-tau, phosphor-p65, phosphor-inhibitor of NF-κB, and brain and serum LPS and TNF-α ↑ brain and serum IL-4 and IL-10 ↑ microbial taxa in oral, fecal and cecal samples	[65]
In vivo; In vitro	Aβ_1–42_-induced AD-like C57BL/6J mice; SH-SY5Y cells	Quercetin-3-O-Glucuronide	50 mg/kg; 20 μM	4 weeks; 45 min	Alleviated cognitive deficit and toxicity	↓ brain insulin resistance ↓ TNF-α, IL-1β, IL-6, and IFN-γ ↑ IL-10 and IL-5 ↓ inflammation-related gut microbiota ↓ Aβ accumulation and tau hyperphosphorylation ↑ CREB and BDNF levels ↑ SCFAs levels ↑ phosphorylation of AKT and MAPK ↓ phosphorylation of JNK and IRS-1	[66]
In vivo	6-month-old male APP/PS1 mice	Curcumin	50 mg/kg/day or 200 mg/kg/day	3 months	Microbiome-targeting therapies for AD	↓ amyloid plaque burden in the hippocampus Bidirectional interactions between curcumin and gut microbiota	[67]
In vivo	D-galactose/AlCl_3_-induced AD male ICR mice	Resveratrol-selenium-peptide nanocomposites	50 mg/kg	16 weeks	Alleviated AD-like pathogenesis and cognitive disorder	↓ Aβ clustering and buildup ↓ Aβ-induced oxidative damage ↓ Aβ aggregate-induced neuroinflammation via the NF-κB/MAPK/Akt pathway ↓ overaction of microglia ↓ gut microbiota disorder ↓ pathogenic bacteria and ↑ beneficial bacteria	[68]
In vivo	5xFAD mice	Prebiotic R13	7.25, 21.8, or 43.6 mg/kg/day	12 to 13 weeks	Mitigated AD pathology	↓ amyloid deposits ↑ *Lactobacillus salivarius* ↓ C/EBPβ/AEP axis ↓ gut leakage and oxidative stress	[69]
In vivo fermentation	Feces from male APP/PS1 mice	Selenium-enriched proteins, particularly H-CVP and H-SBP	The quantities of Se-enriched protein were added based on their Se content (Se = 5 μg/mL)	N/A	Maintained gut health and alleviated cognitive impairment	H-CVP: ↑ *Bacteroidetes* strains H-SBP: ↑ *Firmicutes* and *Lactobacillaceae*	[70]
**Synbiotics**							
In vivo	Transgenic humanized *Drosophila melanogaster* model of AD	3 metabolically active probiotics (*Lactobacillus plantarum* NCIMB 8826, *Lactobacillus fermentum* NCIMB 5221 and *Bifidobacteria longum* spp. *infantis* NCIMB 702255) + a polyphenol rich polyphenol plant extract from the gastrointestinal tonic Triphala	3 × 10^9^ CFU/mL (1:1:1) + 0.5% *w*/*v*	N/A	Delayed AD onset	↑ survival and locomotion ↓ Aβ accumulation and AchE activity ↑ gut–brain-axis pathways and PPARγ	[71]
In vivo	5xFAD mice	*Clostridium sporogenes* + xylan	1 × 10^10^ CFU/day + 1% *w*/*w*	30 days	Improved cognitive and intellectual deficits and ameliorated AD	↓ brain Aβ levels and neuroinflammation ↑ gut barrier integrity and synaptic structure ↑ IPA and IPA-synthesizing bacteria: *Lachnospira* and *Clostridium* ↓ the dominant bacteria in AD: *Aquabacterium*, *Corynebacterium*, and *Romboutsia*	[72]
In vivo	APP transgenic mouse line J20	Vitalon Probiotics (*Bacillus natto*, *Bacillus coagulans*, *Lactobacillus casei*, *Lactobacillus acidophilus*, *Bifidobacterium longum*, *Bifidobacterium breve,* protease, and maltodextrin) + prebiotic (inulin)	4.1 g/kg/day (7:1)	2 months	Alleviated AD-like deficits	↓ Aβ_42_ levels and TNF-α ↑ neurogenesis in the hippocampus ↑ cognitive function	[73]
In vivo	APP/PS1 mice	*Bifidobacterium lactobacillus* and *Lactobacillus acidophilus* + xylo-oligosaccharide	0.5 mL (2.5 × 10^9^ CFU/mL + 0.5 g)	3 times a day for 3 months	Enhanced learning and memory and inhibited AD progression	↓ Aβ deposition and neuroinflammation ↑ PPARs signaling pathways Regulated intestinal microflora	[74]
In vivo	APP/PS1 mice	NMN synbiotics (β-nicotinamide mononucleotide + *Lactobacillus plantarum* + lactulose)	300 mg/kg/day + 10^8^ CFU/mL + 200 mg/kg/day	3 months	Modulated gut microbiota and metabolism	↓ *Firmicutes*/*Bacteroidetes* ratio and microbial diversity ↑ alterations in amino acid and energy metabolic pathways ↑ differential metabolite functions associated with neurotransmitter synthesis and energy metabolism ↓ amyloid plaques formed by Aβ deposition	[75]
In vivo	APP/PS1 mice	Prebiotics (a blend of fibers and plant extracts, including inulin and fruit-oligosaccharides) + probiotics (*Lactobacillus rhamnosus* IMC 501 and *Lactobacillus paracasei* IMC 502)	In diet and water 12 h/day (50:50, bacterial density 10^9^ cells/g)	6 months	Modified pathophysiological hallmarks of AD	↓ Aβ plaques in the CA3 region of the hippocampus ↓ neuronal damage in the CA1 region Modulated astrocyte activation and microglial reactivity	[76]
In vivo	TH-CRE rats infused with adeno-associated virus carrying pseudophosphorylated human tau	ProBiotic-4, comprised of *Bifidobacterium lactis* (50%), *Lactobacillus casei* (25%), *Bifidobacterium bifidum* (12.5%), and *Lactobacillus acidophilus* (12.5%) + prebiotic FOS	3 × 10^9^ CFU/day + 200 mg/kg/day	3 months	Targeted early tau pathology	↓ pretangle tau-related pathology ↑ spatial learning ↓ Iba1, CD68, and GSK-3β ↑ gut microbiome diversity Modulated gut microbiota composition	[77]
In vivo	*App*NL-G-F mice	Lactic acid-producing bacteria (*Lactobacillus plantarum*, *Lactobacillus delbrueckii* subsp. *Bulgaricus*, *Lactobacillus paracasei*, *Lactobacillus acidophilus*, *Bifidobacterium breve*, *Bifidobacterium longum*, *Bifidobacterium infantis*, and *Streptococcus salivarius* subsp. *Thermophilus*) + prebiotic fiber supplement with oligofructose-enriched-inulin	4 × 109 CFU/25 g BW/day + 1.2 mg/20 g BW/day	8 weeks	Showed negligible effects on cognitive abilities	N/A	[78]
**Postbiotics**							
In vitro	Human THP-1 monocytic cells and HL-60 myelomonocytic cells	SCFAs (acetate, propionate, butyrate, valerate, and formate individually or in combination)	Different concentrations, 5–500 μM total concentration (16:8:8:1:1)	15 min	Regulated select immune functions of microglia-like cells	↓ IL-1β, MCP-1, TNF-α and cytotoxins ↓ the phagocytic activity of THP-1 cells ↓ the respiratory burst triggered by fMLP in HL-60 cells and the production of ROS	[79]
In vivo; In vitro	Male APP/PS1 mice; Aβ-stimulated BV2 microglial cells	Acetate	1.5 g/kg/day; 1200 μM	4 weeks; 2 h	Neuroinflammation-alleviating functions for mitigating AD pathology	↓ the phosphorylation of NF-κB p65, ERK, and JNK ↓ CD11b, COX-2 and IL-1β ↑ GPR41	[80]
In vivo; In vitro	5xFAD female mice; HT22 cells	Propionic acid from *Akkermansia muciniphila*	400 mmol/L; 5 mM	2 months; 2 h	Modulated neuronal mitochondrial division and autophagy homeostasis	↓ DRP1 via GPR41 ↑ PINK1/Parkin-mediated mitophagy via GPR43	[81]
In vivo, In vitro	APP/PS1 mice; PC12 cell	SCFAs (sodium acetate, sodium butyrate, and sodium propionate)	Dietary SCFAs; 1 μM/10 μM	9 months; 24 h	Alleviated cognitive deficits and AD pathology	Modulated gut microbiota homeostasis ↓ Aβ plaques and tau hyperphosphorylation ↑ astrocyte-neuron communication (glutamate-glutamine shuttle)	[82]
In vivo	Germ-free APP/PS1 mice	SCFAs (sodium propionate, sodium butyrate, and sodium acetate)	SCFAs (25.9 mM sodium propionate, 40 mM sodium butyrate, and 67.5 mM sodium acetate)	8 weeks	Exacerbated AD pathology	↑ Aβ plaque accumulation ↑ microglial convergence to plaques ↑ ApoE ↓ intracellular Aβ levels in microglia	[83]
In vivo; In vitro	D-galactose-induced aging mice, HT-22 cells	Indoles (indole, IAA, IPA, ILA, and Icld)	Different concentrations of indoles	N/A; 24 h	Neuroprotective effects	↓ oxidative stress, inflammation and neuronal apoptosis induced by H_2_O_2_ ↑ the GPR30/AMP/AMPK/SIRT1 pathway	[84]
In vivo	APP/PS1 male mice	Indoles (mixture of indole, IAA, and IPA)	20 mg/kg/day	4 weeks	Improved gut barrier integrity and cognitive function, and inhibited neuroinflammation	↓ Aβ and hyperphosphorylated tau ↓ TNF-α, IL-6, IL-1β and IL-18 ↑ synaptic plasticity ↑ AhR pathway and ↓ NLRP3 inflammasome	[85]
In vivo	5xFAD male mice	Lysophosphatidylcholine from *Bacteroides ovatus*	20 μM	3 times a week for 4 or 6 weeks	Modulated AD pathologies	↑ synaptic function and cognitive function ↓ Aβ accumulation, gliosis and myelin degeneration ↓ ACSL4 expression via orphan receptor GPR119 to suppress ferroptosis	[86]
In vivo; In vitro	OAβ-injected C57BL6/N male mice; β_23_-overexpressing human embryo kidney 293T cells	Phenyl-γ-valerolactone (PVL)	OAβs preincubated for 15 min with (4′-OH)-PVL (1 µm, 3 µm, 10 µm) at monomeric Aβ_42_:PVL molar concentration ratios ranging from 1:1 to 1:10; different concentrations	24 h; 72 h	Detoxified oAβs and prevented memory impairment	Remodeled preformed oAβ into nontoxic amorphous aggregates ↓ neuroinflammation	[87]
In vitro	Human neuroblastoma SH-SY5Y cells	Exopolysaccharides from *Lactobacillus delbrueckii* ssp. *bulgaricus* B3 and *Lactobacillus plantarum* GD2	100, 250, 500, 1000, or 1250 μg/mL	24 h	Protected against Aβ_1–42_ induced oxidative stress or neurotoxicity	↑ the activities of SOD, CAT and GPx enzymes ↑ ERK1, ERK2, JNK, JUN, NF-κB p65, and p38 ↓ AKT/PKB	[88]
In vivo; In vitro	APP/PS1 mice; HT22 cells	Extracellular vesicles from *Lactobacillus paracasei* (*Lpc*-EV)	2.27 mg/kg/day; Aβ_42_ + *Lpc*-EV (10 μg/mL, final)	1.5 months; 24 h	Reversed Aβ-induced anomalous transcriptional changes	↑ expression of *Bdnf*, *Nt3*, *Nt4/5* and *TrkB* receptor ↑ Aβ-degrading proteases *Mmp-2*, *Mmp-9*, and *Nep* ↑ *MeCP2* and *Sirt1* ↓ Aβ accumulation and neuroinflammatory responses	[89]
In vitro	The mouse microglial cell line (BV-2)	BCM from *Levilactobacillus brevis* CRL 2013, *Lactobacillus delbrueckii* subsp. *lactis* CRL 581, and *Enterococcus mundtii* CRL 35	N/A	N/A	Exhibited antioxidant and anti-inflammatory effects	↓ oxidative stress induced by oAβ_1–42_ ↓ TNF-α, IL-1β, and IL-6 ↓ AchE activity	[90]
In vivo	Male APP/PS1 mice	Tyndallized *Bifidobacterium longum* and *Lactobacillus acidophilus* lysates	120 mg/day	5 times a week for 20 weeks	Disaggregated Aβ_1–40_ aggregates; Slowed down the development of AD	Chelated Zn^2+^ and Cu^2+^ ions ↓ the expression of endogenous human APP transgenic protein and mouse APP gene ↑ mitochondrial LONP1 activity	[91]
In vivo	A polymicrobial mouse model of periodontal disease	Nisin produced by the *Lactococcus lactis*	300 μg/mL, 0.2 mL/day	8 weeks	Mitigated AD-like neuroinflammation triggered by periodontal disease	↓ IL-1β, IL-6, and TNF-α ↓ Aβ_42_, total tau, and phosphorylated tau deposition ↓ microbiome dysbiosis	[92]
In vivo	3-month-old male APP/PS1 mice	Heat-inactivated *Streptococcus thermophilus* MN-ZLW-002	8.33 × 109 CFU/kg/day or 1.67 × 1010 CFU/kg/day	3 months	Alleviated cognitive impairment	↑ colonic propionic acid concentrations ↑ antioxidant defenses in the hippocampus	[93]

Abbreviations: Aβ, amyloid-β; AchE, acetylcholinesterase; ACSL4, acyl-CoA synthetase long-chain family member 4; AD, Alzheimer’s disease; AEP, asparagine endopeptidase; AKT/PKB, protein kinase B; AMPK, AMP-activated protein kinase; ApoE, apolipoprotein E; APP, amyloid precursor protein; APP/PS1, transgenic mouse model expressing human APP and PS1 mutations; BACE1, β-site APP-cleaving enzyme 1; BBB, blood–brain barrier; BCM, bacterial conditioned media; BDNF, brain-derived neurotrophic factor; BW, body weight; CAT, catalase; C/EBPβ, CCAAT/enhancer-binding protein β; CD11b, cluster of differentiation 11b; CFU, colony-forming unit; COS, chitooligosaccharide; COX-2, cyclo-oxygenase-2; CREB, cAMP response element-binding protein; CSP-α, cysteine-string protein α; DRP1, dynamin-related protein 1/mitochondrial fission protein; ERK1, extracellular signal-regulated kinase 1; ERK2, extracellular signal-regulated kinase 2; fMLP, N-formyl-methionyl-leucyl-phenylalanine; FNDC5, fibronectin type III domain-containing protein 5; GABA, γ-aminobutyric acid; GLP-1, glucagon-like peptide-1; GLP-1R, glucagon-like peptide-1 receptor; GLUT1, glucose transporter 1; GLUT3, glucose transporter 3; GPR30, G-protein-coupled receptor 30; GPR41, G-protein-coupled receptor 41; GPR43, G-protein-coupled receptor 43; GPR119, G-protein-coupled receptor 119; GSK-3β, glycogen synthase kinase-3β; GPx, glutathione peroxidase; HPA-axis, hypothalamic-pituitary-adrenal axis; HSP90, heat shock protein 90; H-CVP, Se-enriched cardamine violifolia protein; H-SBP, Se-enriched soybean protein; IAA, indole-3-acetic acid; Iba1, ionized calcium-binding adapter molecule 1; Icld, indole-3-carboxaldehyde; IGF1R, insulin-like growth factor receptor β; ILA, indole-3-lactic acid; IL-1β, interleukin-1β; IL-4, interleukin-4; IL-6, interleukin-6; IL-10, interleukin-10; IL-18, interleukin-18; IFN-γ, interferon-γ; iNOS, inducible nitric oxide synthase; IPA, indole-3-propionic acid; IRS-1, insulin receptor substrate-1; JNK, c-Jun N-terminal kinase; JUN, Jun proto-oncogene, AP-1 transcription factor subunit; LCN-2, lipocalin-2; LONP1, LON peptidase 1; Lpc-EV, *Lactobacillus paracasei*-derived extracellular vesicle; LPS, lipopolysaccharide; MAPK, mitogen-activated protein kinase; MCP-1, monocyte chemoattractant protein-1; MeCP2, methyl-CpG-binding protein 2; Mmp-2, matrix metalloproteinase-2; Mmp-9, matrix metalloproteinase-9; mTOR, mechanistic target of rapamycin; MYD88, myeloid differentiation primary response 88; N/A, not available; NEP, neprilysin; NF-κB, nuclear factor kappa-light-chain-enhancer of activated B cells; NF-L, neurofilament light chain; NLRP3, NOD-like receptor family pyrin domain-containing 3; NOR, novel object recognition; Nrf2, nuclear factor erythroid 2-related factor 2; Parkin, Parkin RBR E3 ubiquitin protein ligase; PINK1, PTEN-induced kinase 1; PPARs, peroxisome proliferator-activated receptors; PPARγ, peroxisome proliferator-activated receptor γ; PS1, presenilin 1; PSD-95, postsynaptic density protein 95; ROS, reactive oxygen species; SCFAs, short-chain fatty acids; SIRT1, sirtuin 1; SOD, superoxide dismutase; TMAO, trimethylamine N-oxide; TLRs, Toll-like receptors; TLR4, toll-like receptor 4; TNF-α, tumor necrosis factor-α; TrkB, tropomyosin receptor kinase B; *w*/*v*, weight/volume; *w*/*w*, weight/weight; 3xTg-AD, triple-transgenic mouse model of Alzheimer’s disease; 4′-OH-PVL, 4′-hydroxy-phenyl-γ-valerolactone; 5-HT, 5-hydroxytryptamine/serotonin; 5xFAD, a transgenic mouse model carrying five familial Alzheimer’s disease mutations; ↓, decrease; ↑, increase.

**Table 2:** The effects and mechanisms of probiotics and synbiotics on Alzheimer’s disease based on clinical trials.

Study Type	Participants	Research Factor	Dose & Duration	Primary Endpoints	Ref.
**Probiotics**					
Multicenter double-blind placebo-controlled RCT	90 patients with mild and moderate AD in Iran (*Lacticaseibacillus rhamnosus* group, *N* = 30; *Bifidobacterium longum* group, *N* = 30; Placebo group, *N* = 30)	*Lacticaseibacillus rhamnosus* HA-114 or *Bifidobacterium longum* R0175	1 × 10^15^ CFU/capsule twice daily for 12 weeks	Boosted cognitive abilities, with the *Bifidobacterium longum* group showing greater enhancements	[94]
Double-blind placebo-controlled RCT	90 patients with mild and moderate AD in Iran (*Lacticaseibacillus rhamnosus* HA-114 group, *N* = 30; *Bifidobacterium longum* R0175 group, *N* = 30; Placebo group, *N* = 30)	*Lacticaseibacillus rhamnosus* HA-114 or *Bifidobacterium longum* R0175	7.5 × 10^9^ CFU/capsule twice daily for 12 weeks	Had beneficial effects on oxidative stress, inflammation, quality of life, and physical activity	[95]
Double-blind active-controlled RCT	32 AD patients in the United States (Treatment group, *N* = 16; Active control group, *N* = 16)	*Bifidobacterium longum* subsp. *infantis* BLI-02, *Bifidobacterium breve* Bv-889, *Bifidobacterium animalis* subsp. *lactis* CP-9, *Bifidobacterium bifidum* VDD088, and *Lactobacillus plantarum* PL-02	(1:1:1:1:1) 1 × 10^10^ CFU/capsule/day for 12 weeks	Enhanced BDNF, ameliorated inflammation and oxidative stress	[96]
**Synbiotics**					
Uncontrolled clinical trial	13 AD patients in Brazil	Kefir-fermented milk	2 mL/kg/day for 90 days	Alleviated cognitive impairment by modulating inflammatory response, oxidative burden, and blood cell injury	[97]
Double-blind placebo-controlled RCT	60 patients with mild to moderate AD in Iran (Intervention group, *N* = 30; Placebo group, *N* = 30)	Gluten-free synbiotic formulation: seven bacterial strains (*Lactobacillus rhamnosus*, *Lactobacillus bulgaricus*, *Lactobacillus casei*, *Lactobacillus acidophilus*, *Bifidobacterium breve*, *Bifidobacterium longum*, and *Streptococcus thermophilus*) + fructo-oligosaccharides	2 capsules/day for 12 weeks (10^9^ CFU of seven bacterial strains/capsule)	No detectable cognitive enhancement was observed	[98]

Abbreviations: AD, Alzheimer’s disease; BDNF, brain-derived neurotrophic factor; CFU, colony-forming unit; RCT, randomized controlled trial.

**Table 3:** The effects and mechanisms of prebiotics and postbiotics on Alzheimer’s disease from epidemiological studies.

Study Type	Participants	Research Factor	Primary Endpoints	Ref.
**Prebiotics**				
Cohort study	1837 elderly (≥65 years) northern Manhattan residents who were dementia-free at baseline from the multi-ethnic Washington Heights-Inwood Columbia Aging Project	Fructan	Every extra gram of daily fructan intake was associated with 24% lower risk for AD (95% *CI*: 0.60–0.97; *p* = 0.03)	[101]
Cross-sectional study	1788 participants from the NHANES for the years 2011–2014	Nonfood prebiotics: glucan, gum arabic, inulin, chicory, resistant starch, psyllium, resveratrol, oligofructose, oligosaccharides, lactulose, and other prebiotic supplements	In elderly men from the USA, nonfood pro- or prebiotic intake was associated with higher comprehensive cognitive function (*β* = 0.64, 95% *CI*: 0.08–1.19) and lower risk of cognitive impairment (*OR* = 0.08, 95% *CI*: 0.02–0.29) compared with those who did not consume pro- or prebiotic	[102]
Cross-sectional study	1704 participants from the NHANES for the years 2011–2014	Nonfood prebiotics: acacia gum, chicory, glucan, wheat dextrin, inulin, lactulose, resistant starch, polydextrose, oligofructose, oligosaccharides, psyllium, and other prebiotic supplements	In participants with cardiovascular disease history, prebiotic intake was associated with higher global cognition (*β* = 0.24, 95% *CI*: 0.03–0.46) and CERAD-DRT (*β* = 0.35, 95% *CI*: 0.02–0.68) z-score compared with those without prebiotic intake	[103]
**Postbiotics**				
Cross-sectional study	Cases with positive amyloid PET scans for biomarkers of AD (*N* = 19) and healthy controls without AD (*N* = 19) in China	Propionic acid	Lower serum and fecal propionic acid concentrations were associated with AD (*p* < 0.0002)	[81]
Cross-sectional study	Cases diagnosed with AD (*N* = 29) and age-matched normal controls (*N* = 29) in China	LPC	Lower serum and fecal LPC concentrations were associated with AD (*p* < 0.05)	[86]

**Abbreviations:** AD, Alzheimer’s disease; *CI*, confidence interval; CSF, cerebrospinal fluid; LPC, lysophosphatidylcholine; MCI, mild cognitive impairment; PET, positron emission tomography.

## Data Availability

No new data were created or analyzed in this study.

## Abbreviations

The following abbreviations are used in this manuscript: 5-HT, 5-hydroxytryptamine/serotonin; 3xTg-AD, triple-transgenic mouse model of Alzheimer’s disease; 4′-OH-PVL, 4′-hydroxy-phenyl-γ-valerolactone; 5xFAD, transgenic mouse model carrying five familial Alzheimer’s disease mutations; Aβ, amyloid-β; AchE, acetylcholinesterase; ACSL4, acyl-CoA synthetase long-chain family member 4; AD, Alzheimer’s disease; AEP, asparagine endopeptidase; AhR, aryl hydrocarbon receptor; AKT, protein kinase B; AlCl_3_, aluminum chloride; AMPK, AMP-activated protein kinase; ApoE, apolipoprotein E; APP, amyloid precursor protein; APPswe, amyloid precursor protein Swedish mutation; APP/PS1, transgenic mouse model expressing human APP and PS1 mutations; APOE4, apolipoprotein ε4 allele; ATP, adenosine triphosphate; BACE1, β-site APP-cleaving enzyme 1; BBB, blood–brain barrier; BCM, bacterial conditioned media; BDNF, brain-derived neurotrophic factor; BW, body weight; C/EBPβ, CCAAT/enhancer-binding protein β; CAT, catalase; CD11b, cluster of differentiation 11b; CD68, cluster of differentiation 68; CFU, colony-forming unit; CI, confidence interval; COS, chitooligosaccharide; COX-2, cyclo-oxygenase-2; CREB, cAMP response element-binding protein; CSF, cerebrospinal fluid; CSP-α, cysteine-string protein α; DOP, *dendrobium officinale* polysaccharides; DRP1, dynamin-related protein 1/mitochondrial fission protein; DSM, Deutsche Sammlung von Mikroorganismen; E4FAD, APOE4 mouse line; EPSs, exopolysaccharides; ERK, extracellular signal-regulated kinase; ERK1, extracellular signal-regulated kinase 1; ERK2, extracellular signal-regulated kinase 2; EVs, extracellular vesicles; fMLP, N-formyl-methionyl-leucyl-phenylalanine; FNDC5, fibronectin type III domain-containing protein 5; FOS, fructo-oligosaccharides; GABA, γ-aminobutyric acid; GLP-1, glucagon-like peptide-1; GLP-1R, glucagon-like peptide-1 receptor; GLUT1, glucose transporter 1; GLUT3, glucose transporter 3; GOS, galacto-oligosaccharides; GPR30, G-protein-coupled receptor 30; GPR41, G-protein-coupled receptor 41; GPR43, G-protein-coupled receptor 43; GPR119, G-protein-coupled receptor 119; GPx, glutathione peroxidase; GSK-3β, glycogen synthase kinase-3β; H-CVP, Se-enriched cardamine violifolia protein; HO-1, heme oxygenase-1; HPA-axis, hypothalamic-pituitary-adrenal axis; H-SBP, Se-enriched soybean protein; HSP90, heat shock protein 90; IAA, indole-3-acetic acid; Iba1, ionized calcium-binding adapter molecule 1; Icld, indole-3-carboxaldehyde; IFN-γ, interferon-γ; IGF1R, insulin-like growth factor receptor β; ILA, indole-3-lactic acid; IL-1β, interleukin-1β; IL-4, interleukin-4; IL-5, interleukin-5; IL-6, interleukin-6; IL-10, interleukin-10; IL-18, interleukin-18; iNOS, inducible nitric oxide synthase; IPA, indole-3-propionic acid; IRS-1, insulin receptor substrate-1; ISAPP, International Scientific Association for Probiotics and Prebiotics; JNK, c-Jun N-terminal kinase; JUN, Jun proto-oncogene, AP-1 transcription factor subunit; KOS, κ-carrageenan oligosaccharides; LCN-2, lipocalin-2; LONP1, LON peptidase 1; LPC, lysophosphatidylcholine; Lpc-EV, *Lactobacillus paracasei*-derived extracellular vesicles; LPS, lipopolysaccharide; MAPK, mitogen-activated protein kinase; MCI, mild cognitive impairment; MCP-1, monocyte chemoattractant protein-1; MDA, malondialdehyde; MeCP2, methyl-CpG-binding protein 2; MMP-2, matrix metalloproteinase-2; MMP-9, matrix metalloproteinase-9; MMSE, Mini-Mental State Examination; MOS, mannan oligosaccharide; mTOR, mechanistic target of rapamycin; MYD88, myeloid differentiation primary response 88; N/A, not available; NCIMB, National Collection of Industrial, Food and Marine Bacteria; NEP, neprilysin; NF-κB, nuclear factor-κB; NF-L, neurofilament light chain; NLRP3, NOD-like receptor family pyrin domain containing 3; NMN, β-nicotinamide mononucleotide; NOR, novel object recognition; Nrf2, nuclear factor erythroid 2-related factor 2; Nt3, neurotrophin 3; Nt4/5, neurotrophin 4/5; oAβ, Aβ oligomer; Parkin, Parkin RBR E3 ubiquitin protein ligase; PET, positron emission tomography; PI3K, phosphoinositide 3-kinase; PINK1, PTEN-induced kinase 1; PPARγ, peroxisome proliferator-activated receptor gamma; PPARs, peroxisome proliferator-activated receptors; PPSP, probiotics, prebiotics, synbiotics, and postbiotics; PS1, presenilin 1; PSD-95, postsynaptic density protein 95; PVL, phenyl-γ-valerolactone; RCT, randomized controlled trial; ROS, reactive oxygen species; SAMP8, senescence-accelerated prone 8; SCFAs, short-chain fatty acids; SIRT1, sirtuin 1; SOD, superoxide dismutase; Tg-APP/PS1, transgenic APP/PS1; TH-CRE, tyrosine hydroxylase-Cre; TLR4, toll-like receptor 4; TLRs, Toll-like receptors; TMAO, trimethylamine N-oxide; TNF-α, tumor necrosis factor-α; TrkB, tropomyosin receptor kinase B; *w*/*v*, weight/volume; *w*/*w*, weight/weight.
